# A CRY1 Interactor eIF3G1 Negatively Regulates Root Growth Under Blue Light in *Arabidopsis*

**DOI:** 10.3390/plants15111682

**Published:** 2026-05-29

**Authors:** Xiali Chen, Jinyu Pang, Lingling Liu, Wanqi Li, Yan Zhang, Juan Feng, Xian Xiang, Qiyao Wu, Rongbin Fan, Lina Qu, Jun Su, Qin Wang, Chentao Lin, Zonghua Wang, Guifang Lin

**Affiliations:** 1 College of Life Sciences, Fujian Agriculture and Forestry University, Fuzhou 350002, China; chenxiali2022@163.com (X.C.); pangjiny@126.com (J.P.); lingl006@foxmail.com (L.L.); 15060633995@163.com (W.L.); 14727643860@163.com (Y.Z.); fengjuan20030122@163.com (J.F.); xianxiang92@foxmail.com (X.X.); frb0606@foxmail.com (R.F.); 2 Basic Forestry and Plant Proteomics Research Center, Haixia Institute of Science and Technology, Fujian Agriculture and Forestry University, Fuzhou 350002, China; fafuqiyaowu@163.com (Q.W.); junsu@fafu.edu.cn (J.S.); qinwangcry@163.com (Q.W.); chentaolin163@163.com (C.L.); 3 College of Agriculture, Fujian Agriculture and Forestry University, Fuzhou 350002, China; qulina@fafu.edu.cn; 4 Fujian Universities Key Laboratory for Plant-Microbe Interaction, College of Life Sciences, Fujian Agriculture and Forestry University, Fuzhou 350002, China; 5 Fuzhou Institute of Oceanography, Minjiang University, Fuzhou 350108, China

**Keywords:** eIF3G1, CRY1, blue light, root growth, root meristem, *Arabidopsis thaliana*

## Abstract

Plants perceive light signals through photoreceptors such as CRY1 to regulate growth and development. It is well-known that *Arabidopsis* CRY1 is a nucleocytoplasmic protein that mediates light inhibition of hypocotyl elongation in the nucleus, but the mechanisms by which CRY1 regulates root growth and functions in the cytoplasm remain poorly understood. Here, we identified eIF3G1, a subunit of the eukaryotic translation initiation factor 3 (eIF3) complex, as a CRY1-interacting protein associated with light-regulated root development. Under blue light, *eif3g1* mutants showed longer primary roots, whereas *eIF3G1* overexpression reduced root elongation, accompanied by corresponding changes in root apical meristem size. Differential irradiation experiments indicated that shoot illumination is required for *eIF3G1*-dependent root phenotypes. Transcriptome analysis revealed changes in translation-related and light-responsive genes in response to *eIF3G1* perturbation. Comparison with the *cry1* transcriptome revealed overlapping differentially expressed genes, including *BIC1* and *BIC2*, and the *bic1 bic2* double mutant showed reduced root elongation. Together, these findings identify eIF3G1 as a CRY1-interacting factor that contributes to the shoot-dependent regulation of root growth under blue light, suggesting that eIF3G1 may be associated with the CRY1-dependent shoot-to-root regulation of root growth.

## 1. Introduction

Light is not only an energy source, but also a key environmental signal for plant growth and development. To perceive light, plants have evolved multiple classes of photoreceptors that regulate photomorphogenesis and skotomorphogenesis [1]. Among these, blue-light receptors, cryptochromes (CRYs, including CRY1 and CRY2 in *Arabidopsis*), play important roles in mediating diverse photoresponses, such as hypocotyl elongation, floral transition, and circadian rhythm regulation [2,3,4,5]. The molecular mechanisms underlying these processes are largely mediated through interactions between CRYs and their interacting proteins [6]. To date, more than 80 interacting proteins have been identified [7], which associate with CRYs in both blue light-dependent and -independent manners. Most of the known CRY interactors are nuclear proteins that regulate transcription and chromatin modification, mRNA splicing, mRNA methylation, and proteolysis [6,8,9,10,11]. In contrast to CRY2, which acts exclusively in the nucleus, CRY1 is a nucleocytoplasmic protein that is known to act in both the nucleus and cytoplasm [12,13,14]. However, how CRY1 regulates root development and functions in the cytoplasm remain poorly understood.

Roots typically develop in dark soil environments, yet their growth is profoundly influenced by light perceived in either shoots or roots [15]. Shoot-perceived light signals are transmitted to roots via mobile factors such as transcription factors, hormones, and sugars. For example, the transcription factor ELONGATED HYPOCOTYL 5 (HY5) moves from the shoots to roots to coordinate root development and nutrient acquisition [16], while light also regulates root elongation through the COP1-dependent modulation of auxin transport [17]. In addition, photosynthetically produced sucrose transported from the shoots to roots acts together with light to promote root growth [18,19]. Besides long-distance signals, roots can directly perceive light through stem-piped or locally exposed illumination [20,21]. Within these regulatory networks, photoreceptors serve as the primary molecular sensors that initiate light-driven root responses [19,22]. Among them, the blue-light-sensing CRYs play indispensable roles. In darkness, CRY2 has been reported to inhibit root growth by interacting with FORKED- LIKE 1 (FL1) and FL3 to suppress apical meristem activity [23]. Under blue light, CRY1 is known to promote root development, at least in part, through modulating auxin-related pathways [24,25]. However, the molecular mechanisms by which CRY1 regulates root development remain largely unclear.

The eukaryotic translation initiation factor 3 subunit G (eIF3g) is a conserved component of the eIF3 complex that functions in translation initiation and reinitiation through its RNA recognition motif (RRM) [26,27,28]. In addition to its general role in translation initiation, eIF3g functions as a key regulator of transcript-selective translation by binding to specific mRNA sequence features, thereby contributing to gene-specific regulation under stress conditions [29,30]. While studies in other eukaryotes have implicated eIF3g in diverse biological processes and disease-related pathways [31,32,33,34,35,36,37], its specific roles and regulatory mechanisms in plants remain comparatively limited. In plants, eIF3g has been associated with environmental adaptation and stress-responsive translational regulation. *Arabidopsis* encodes two homologs of eIF3g, eIF3G1 and eIF3G2 [38], among which eIF3G1 has been reported to interact with the heat shock factor HSFB1 to coordinate growth and stress responses [39]. Consistently, heterologous expression of wheat TaeIF3g in *Arabidopsis thaliana* confers enhanced tolerance to drought, heat, and osmotic stress [40], suggesting a conserved role for eIF3g proteins in stress adaptation. Notably, wheat TaeIF3g proteins exhibit approximately 70% identity compared with *Arabidopsis* eIF3G1 at the amino acid level [41], further supporting their evolutionary conservation. Based on its identification in our IP-MS screening dataset as a CRY1-interacting candidate and its reported association with translational regulation, eIF3G1 was prioritized for further validation among the identified interactors. The CRY1–eIF3G1 interaction was subsequently confirmed using multiple independent assays, supporting a physical association.

In this study, we investigated the physical and functional relationship between eIF3G1 and the blue-light receptor CRY1 in *Arabidopsis*. We identified eIF3G1 as a CRY1-interacting protein, and through genetic analysis, found that it negatively affects blue light-induced root development and exhibits phenotypes opposite to those of *cry1* mutants. Using differential illumination experiments, we further show that *eIF3G1* participates in the shoot-dependent regulation of root growth. By integrating transcriptome profiling and comparative analysis with *cry1* data, we identified overlapping differentially expressed genes including *Blue-light Inhibitors of Cryptochromes 1* and *2* (*BIC1* and *BIC2*). Together, these findings suggest that eIF3G1 may function as a component associated with cryptochrome signaling in the regulation of root development.

## 2. Results

### 2.1. eIF3G1 Physically Interacts with CRY1 in the Cytoplasm

**Figure 1:**
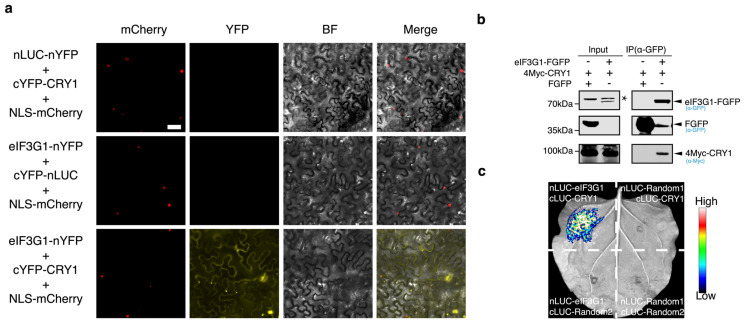
eIF3G1 physically interacts with CRY1 in the cytoplasm of *N. benthamiana*. (**a**) BiFC assay revealing the interaction between eIF3G1 and CRY1 in the cytoplasm of *N. benthamiana* leaf cells. Scale bar = 20 μm. Red and yellow fluorescence represent the mCherry-tagged nuclear localization signal (NLS) and the reconstituted YFP signal, respectively. (**b**) Co-IP analysis of eIF3G1 and CRY1 in *N. benthamiana*. Total protein extracts were immunoprecipitated and analyzed by immunoblotting with the indicated antibodies. The asterisk (*) indicates non-specific bands. Molecular weights in kDa are indicated on the left. (**c**) Qualitative observation of the Split-LUC assay confirming the interaction between eIF3G1 and CRY1 in *N. benthamiana* leaves. Data shown are from one representative experiment independently repeated three times with similar results.

To identify novel interacting proteins of CRY1, we reanalyzed our previously generated co-immunoprecipitation coupled with mass spectrometry (IP-MS) data and identified eIF3G1 as a candidate interactor. To validate this interaction in vivo, we first performed bimolecular fluorescence complementation (BiFC) assays in *Nicotiana benthamiana*. Co-expression of eIF3G1-nYFP and cYFP-CRY1 reconstituted YFP fluorescence, whereas no detectable signals were observed in the negative controls (Figure 1a). Notably, the BiFC signal was clearly observed in the cytoplasm (Figure 1a). In addition, fluorescence observation of stable transgenic *Arabidopsis* seedlings expressing eIF3G1-Flag-GFP showed that eIF3G1 was predominantly localized in the cytoplasm of root tip cells, consistent with the transient expression results observed in *Nicotiana benthamiana* (Figures S1 and S2). The physical interaction was further validated using co-immunoprecipitation (Co-IP) assays. eIF3G1 was specifically co-immunoprecipitated with CRY1 from plant protein extracts (Figure 1b and Figure S3), demonstrating their interaction at the biochemical level. Consistently, split-luciferase complementation (Split-LUC) assays revealed strong chemiluminescence signals in leaves co-expressing cLUC-CRY1 and eIF3G1-nLUC (Figure 1c), supporting an in planta association between CRY1 and eIF3G1. Collectively, these results suggest that CRY1 physically interacts with eIF3G1 in the cytoplasm. This cytoplasmic localization may be relevant to the cytoplasmic functions previously reported for CRY1.

### 2.2. eIF3G1 Negatively Regulates Primary Root Elongation Under Blue Light

CRY1 is a primary blue light photoreceptor that mediates various aspects of photomorphogenesis, including the inhibition of hypocotyl elongation and the regulation of root growth [14,24]. Given the physical interaction between CRY1 and eIF3G1, we investigated whether *eIF3G1* is associated with seedling development. We analyzed hypocotyl and primary root elongation in a T-DNA insertion mutant (*eif3g1*, SALK_029432) and two independent *eIF3G1* overexpression lines (*OE#4* and *OE#8*; Figure S1) under both dark and blue light conditions using wild-type (WT) and *cry1* mutants as controls. The *eif3g1* mutant has previously been characterized [39], and its genotype was further validated in this study (Figure S4).

**Figure 2:**
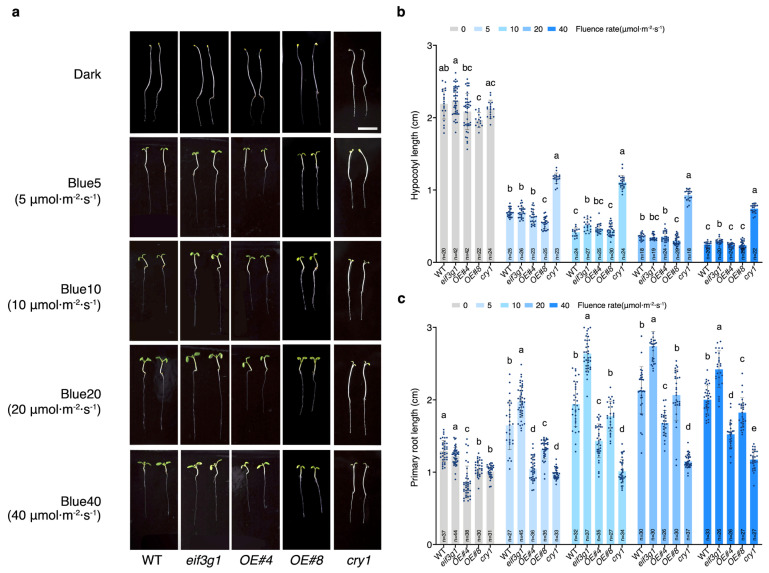
*eIF3G1* negatively regulates primary root elongation under blue light. (**a**) Representative images of root growth in WT, *eif3g1* mutant, *OE#4*, *OE#8*, and *cry1* mutant seedlings grown on 1/2 MS medium for 5 days under continuous darkness or different blue light conditions, scale bar = 1 cm. (**b**,**c**) Quantification of (**b**) hypocotyl length and (**c**) primary root length of WT, *eif3g1*, *OE#4*, *OE#8*, and *cry1* seedlings under the indicated growth conditions. Data are presented as grouped bar plots, with bars representing the mean ± SD, and individual dots indicating biological replicates. Data shown are from one representative experiment independently repeated at least three times with similar results. The number of biological replicates (*n*) is indicated on each bar. Different lowercase letters indicate significant differences among genotypes within the same light condition (*p* < 0.05, one-way ANOVA followed by Tukey’s multiple comparisons test).

For hypocotyl elongation, most genotypes did not differ significantly from WT in darkness or under blue light conditions (*p* < 0.05; Figure 2a,b). *OE#8* exhibited significantly shorter hypocotyls in darkness and under 5 and 20 μmol·m^−2^·s^−1^ blue light, whereas *eif3g1* displayed significantly elongated hypocotyls at 10 and 40 μmol·m^−2^·s^−1^ blue light. *OE#4* remained comparable to WT across all tested conditions. As expected, *cry1* consistently exhibited significantly elongated hypocotyls relative to WT under all tested blue-light conditions.

In contrast to hypocotyl growth, primary root elongation exhibited a more consistent phenotype among the tested genotypes (Figure 2a,c). In darkness, no significant difference in primary root length was observed between the *eif3g1* mutant and WT. However, under blue light, *eif3g1* seedlings developed significantly longer primary roots than WT across various light intensities (5, 10, 20, and 40 μmol·m^−2^·s^−1^; *p* < 0.05), with differences ranging from approximately 0.3 to 0.8 cm. Conversely, both independent *eIF3G1* overexpression lines showed significantly reduced primary root elongation under both dark and blue-light conditions. This phenotype may partially reflect effects associated with constitutive overexpression of *eIF3G1*, although this possibility was not directly tested in this study. In contrast, the *eif3g1* mutant exhibited enhanced root elongation specifically under blue light, but not in darkness. Together, these results suggest that eIF3G1 has a negative effect on primary root elongation, particularly under blue light conditions.

### 2.3. eIF3G1 Is Associated with Root Apical Meristem Size Under Blue Light

**Figure 3:**
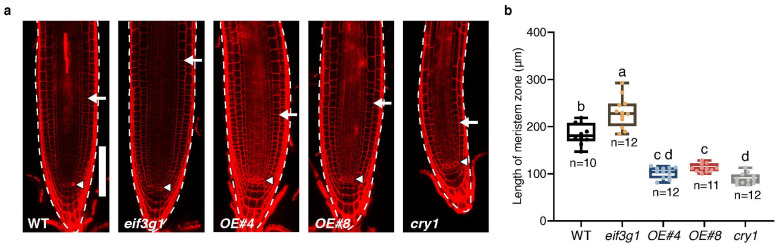
*eIF3G1* is associated with root apical meristem size under blue light. (**a**) Confocal images of root tips from 5-day-old seedlings. White arrows indicate the boundary between the root meristem and transition zone, and arrowheads indicate the quiescent center (QC). Scale bar = 100 μm. (**b**) Quantification of meristem zone length in 5-day-old seedlings shown in (**a**). Data are presented as mean ± SD with individual data points (*n* ≥ 10). Data shown are from one representative experiment independently repeated three times with similar results. Different lowercase letters indicate significant differences determined using one-way ANOVA followed by Tukey’s multiple comparisons test (*p* < 0.05).

Given the observed alterations in primary root elongation, we next investigated whether the root apical meristem (RAM) size was affected under blue light conditions. Root tips of 5-day-old seedlings were stained with propidium iodide (PI) and visualized by confocal microscopy. In WT seedlings, the meristematic zone and transition zone were clearly distinguishable (Figure 3a). Compared with WT, *eif3g1* mutants displayed an expanded meristematic region, whereas *OE#4* and *OE#8* showed a visibly reduced meristematic zone. Quantification of meristem length confirmed that RAM length was significantly increased in *eif3g1* mutants and reduced in overexpression lines relative to WT (Figure 3b). The *cry1* mutant also exhibited altered meristem length consistent with its root growth phenotype. The RAM size changes were consistent with the differences observed in primary root elongation. These results indicate that eIF3G1 is associated with changes in root apical meristem size and root growth under blue light conditions.

### 2.4. Shoot Illumination Is Required for eIF3G1-Dependent Root Growth Regulation

**Figure 4:**
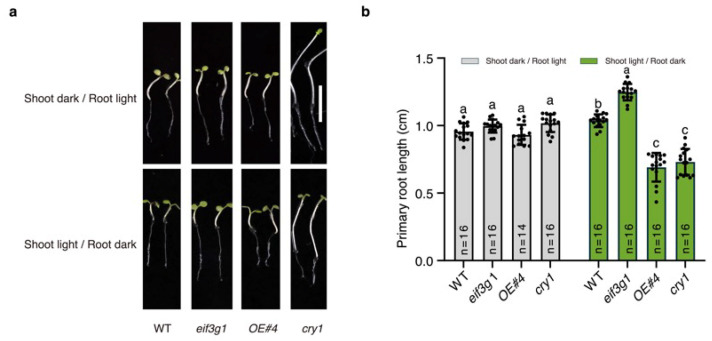
Shoot illumination is required for *eIF3G1*-dependent root growth regulation. (**a**) Representative phenotypes of seedlings grown under spatially separated light conditions: shoots are exposed to light and roots are in darkness (Shoot light/Root dark), or shoots are in darkness and roots are exposed to light (Shoot dark/Root light). Scale bar = 1 cm. (**b**) Quantification of primary root length under the indicated shoot/root illumination conditions. Data are presented as mean ± SD (*n* ≥ 14). Different lowercase letters indicate significant differences determined using one-way ANOVA followed by Tukey’s multiple comparison test (*p* < 0.05).

As roots typically develop in soil, whereas light signals can be sensed by both shoots and roots, we investigated shoot–root communication under different illumination conditions. We employed a separate illumination system, as previously described [21], to evaluate the effects of selective tissue irradiation on 3-day-old seedlings. Primary root length data were analyzed separately within each illumination condition using one-way ANOVA followed by Tukey’s multiple comparison test (*n* ≥ 14 seedlings per condition; Figure 4). We focused on whether the root phenotypes observed in *eif3G1* depend on direct root exposure to blue light or on signals derived from the shoot. We found that no significant differences in primary root length were observed among the WT, *eif3g1* mutants, *eIF3G1* overexpression lines, and *cry1* mutants when the roots were exposed to blue light (Shoot dark/Root light). In contrast, when the shoots were illuminated with blue light while the roots were shielded from light (Shoot light/Root dark), the *eif3g1* mutants developed significantly longer primary roots (*p* < 0.05), whereas the *OE#4* and *cry1* mutants exhibited reduced root growth compared with WT (*p* < 0.05). These results are consistent with phenotypes previously observed under whole seedling exposure to blue light. Together, these findings suggest that shoot light perception is associated with differences in primary root elongation in *eIF3G1*-related lines under blue light. These data suggest that eIF3G1 may be associated with shoot-light-dependent root growth responses, potentially involving shoot-to-root signaling, as previously proposed for CRY1 [24].

### 2.5. Transcriptome Profiling Reveals Shared and Genotype-Specific Transcriptional Changes upon Altered eIF3G1 Expression

To explore the molecular basis underlying the contrasting root growth phenotypes associated with altered *eIF3G1* expression, we performed RNA-Seq analysis using roots from 5-day-old WT, *eif3g1*, and *OE#4* seedlings. Whole seedlings were exposed to 20 μmol m^−2^ s^−1^ blue light, a condition under which clear root growth differences among the genotypes were observed. Although blue light perception in the shoot is required for *eIF3G1*-mediated root regulation, the resultant phenotypic difference is primarily manifested in the root tissue. To reduce the influence of transcriptional changes associated with primary light responses in the shoot, we performed RNA-Seq on root tissues. This approach was used to assess local transcriptional responses associated with root growth differences in eIF3G1-related lines.

**Figure 5:**
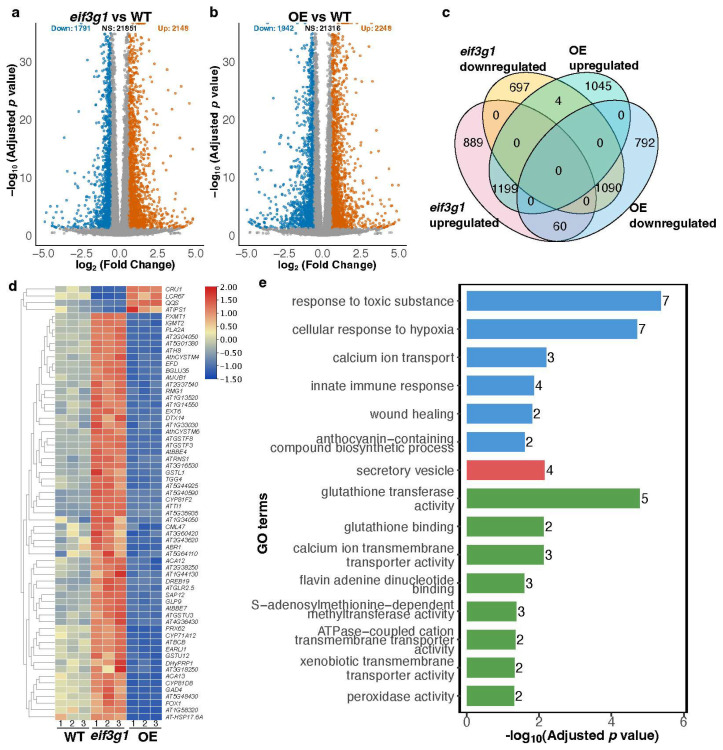
Transcriptomic analysis of the *eif3g1* mutant and *eIF3G1* overexpression lines. (**a**,**b**) Volcano plots showing DEGs in *eif3g1* vs. WT (**a**) and *OE#4* vs. WT (**b**). The *x*-axis represents log_2_ fold change and the *y*-axis represents −log_10_ (adjusted *p* value). Upregulated genes are shown in orange, downregulated genes in blue, and non-significant genes in gray. The numbers of upregulated, downregulated, and non-significant genes are indicated above each plot. (**c**) Venn diagram illustrating the overlap between upregulated and downregulated DEGs in the *eif3g1* mutant and *OE#4*. (**d**) Heatmap displaying the expression patterns of DEGs showing opposite regulation between the *eif3g1* mutant and *OE#4* across the WT, *eif3g1*, and *OE#4* samples. Color scale indicates normalized expression levels (red, higher expression; blue, lower expression). (**e**) GO enrichment analysis of genes downregulated in *OE#4* but upregulated in the *eif3g1* mutant. The bar plot shows significantly enriched GO terms in the biological process (blue), cellular component (red), and molecular function (green) categories. The *x*-axis represents −log_10_ (adjusted *p* value), and the numbers beside bars indicate gene counts associated with each term.

Principal component analysis (PCA) revealed distinct genotype-dependent clustering, with PC1 and PC2 accounting for 59.1% and 33.6% of the total variance, respectively (Figure S5). Notably, while the WT was positioned on the left side of the PC1 axis, both the *eif3g1* mutant and *OE#4* line shifted in the same direction toward the right. Differential expression analysis identified 3939 and 4190 differentially expressed genes (DEGs) in *eif3g1* and *OE#4* plants relative to WT, respectively (Figure 5a,b; Tables S1 and S2). A substantial proportion of DEGs were shared between the two genotypes and showed the same direction of regulation (Figure 5c), including 1199 genes that were commonly upregulated and 1090 genes that were commonly downregulated in both the *eif3g1* and *OE#4* plants relative to WT. Gene Ontology (GO) enrichment analysis revealed that these commonly upregulated genes were primarily associated with light-responsive pathways, photosynthesis, hormone signaling, and stress-related processes (Figure S6). Conversely, the commonly downregulated genes were significantly enriched in ribosome biogenesis, translational processes, DNA replication, chromatin organization, and cell cycle progression (Figure S6). The downregulation of genes involved in ribosome biogenesis and translational processes may reflect an imbalance of the translation initiation complex upon *eIF3G1* perturbation.

In addition to this shared module, a smaller subset of genes exhibited opposite regulation between *eif3g1* and *OE#4* (Figure 5c,d; Table S3). Only four genes were downregulated in *eif3g1* but upregulated in *OE#4*, whereas 60 genes showed the inverse pattern. GO enrichment analysis of these 60 genes revealed significant overrepresentation of categories related to redox homeostasis, detoxification processes, calcium ion transport, and innate immune responses (Figure 5e).

Detailed transcriptomic profiling (Figure 5d; Table S3) identified several antioxidant enzymes within this group, such as glutathione S-transferases (*ATGSTF3*, *ATGSTF8*, *GSTU12*) and peroxidases (*PRX62*, *AT4G36430*). The induction of these genes is often associated with cellular redox perturbations, which can influence the transition between cell proliferation and differentiation in the root tip [21,42]. Furthermore, transcription factors known to modulate growth-to-stress transitions, such as *AtJUB1* [43], were specifically elevated in the *eif3g1* mutant. Conversely, genes showing a positive correlation with *eIF3G1* levels, such as the orphan gene *QQS* and the phosphate-responsive *ATIPS1*, are involved in metabolic regulation and nutrient signaling, respectively [44,45]. Together, the altered expression of these candidates suggests that changes in redox and stress-responsive pathways may be associated with eIF3G1-related divergent root growth phenotypes.

### 2.6. Identification of BIC1 and BIC2 as Shared Transcriptional Targets of CRY1 and eIF3G1 in Roots

**Figure 6:**
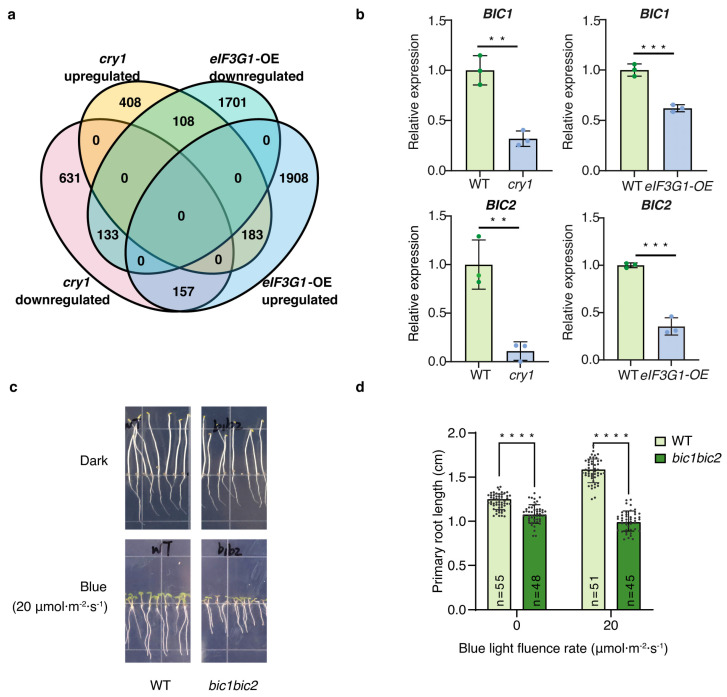
*BIC1* and *BIC2* are shared downstream targets of *CRY1* and *eIF3G1*, and their loss results in reduced primary root elongation. (**a**) Venn diagram showing the overlap of differentially expressed genes identified from RNA-Seq analyses of the *cry1* mutant and *eIF3G1-OE#4* line under blue light. Numbers indicate gene counts within each category. (**b**) Transcript abundance (FPKM) of *BIC1* and *BIC2* in WT versus *cry1* and *eIF3G1-OE#4* seedlings under blue light. The *cry1* mutant and *eIF3G1-OE#4* line were each compared with their respective WT controls from the same batch. Data are presented as mean ± SD (*n* = 3). Statistical significance was determined using two-tailed unpaired Student’s *t*-tests (** *p* < 0.01, *** *p* < 0.001). Each pair was compared independently. (**c**) Representative images of five-day-old WT and *bic1bic2* seedlings grown on 1/2 MS medium under continuous darkness or the indicated blue light conditions, Scale bar = 1 cm. (**d**) Quantification of primary root length under the indicated light conditions. Statistical analysis was performed using two-way ANOVA followed by Tukey’s multiple comparisons test. Asterisks indicate significant differences between WT and *bic1bic2* within each light condition (**** *p* < 0.0001). Data are represented as mean ± SD (*n* ≥ 46).

To explore potential transcriptional coordination between *eIF3G-* and *CRY1-*mediated root development under blue light, we generated a root transcriptome dataset from *cry1* seedlings grown under blue light and compared it with the RNA-Seq data of *eIF3G1 OE#4* plants, as both genotypes exhibit significantly shorter primary roots. Differential expression analysis identified a total of 1620 DEGs in *cry1* roots relative to WT (Figure S7). Venn diagram analysis revealed that 316 genes were differentially expressed in both *cry1* and *OE#4* roots compared with WT (Figure 6a; Table S5), indicating a partial overlap in their transcriptional responses.

GO enrichment of these co-regulated DEGs showed that repressed genes were primarily involved in biological processes, including response to light stimulus, response to heat, and response to auxin (Tables S6 and S7). Conversely, upregulated genes were enriched in photosynthesis and cold acclimation. These results suggest that eIF3G1 and CRY1 may coordinately modulate root growth by impacting auxin signaling and photosynthetic feedback. Interestingly, although HY5 is a key mobile signal that coordinates shoot-to-root growth through a transcriptional auto-activation loop [16], its expression was significantly downregulated only in *cry1* roots and remained unaffected in the *OE#4* transcriptome (Tables S2 and S4). The lack of significant changes in local root *HY5* transcript levels does not provide evidence for its involvement in the observed transcriptional changes associated with root growth inhibition in *eIF3G1*-overexpressing plants.

Among the 316 shared genes, we identified *BIC1* and *BIC2*, two important regulators of blue-light signaling. Transcriptomic profiling confirmed that both *BIC1* and *BIC2* were significantly downregulated in *cry1* and *OE#4* (Tables S2 and S4; Figure 6b). In the *eif3g1* mutant, *BIC2* expression was moderately reduced, whereas *BIC1* expression did not exhibit a statistically significant change (Table S1). CRY1 is known to regulate *BIC1* and *BIC2* expression through COP1-dependent pathways, and the *bic1bic2* double mutant exhibits shortened hypocotyls [46]. To determine whether reduced expression of *BIC1* and *BIC2* also affects root development, we analyzed primary root elongation in the *bic1bic2* double mutant under both blue light and dark conditions. Phenotypic analysis revealed that *bic1bic2* seedlings exhibited significantly shorter primary roots compared with WT under both conditions (Figure 6c,d). These results indicate that *BIC1* and *BIC2* contribute to primary root elongation. Collectively, the consistent downregulation of *BIC1* and *BIC2* in *OE#4* and *cry1* supports their identification as shared differentially expressed genes of *eIF3G1* and *CRY1* in roots. However, whether they function as downstream effectors, and how their expression is regulated within this pathway, remain to be determined.

## 3. Discussion

Photoreceptors serve as the primary molecular sensors for light-regulated development, mediating diverse photoresponses such as photomorphogenesis and environmental adaptation through the assembly of protein–protein interaction complexes. As blue light receptors, CRYs have been identified by more than 80 interacting proteins [7] and are still increasing, but the full complexity of the CRY signaling network remains largely unexplored. In this study, we identified eIF3G1, a key component of the eIF3 complex containing a conserved RRM motif [26], as a novel cytoplasmic interactor of the blue light receptor CRY1. Our results suggest that the expression balance of eIF3G1 may be important for translational regulation, consistent with its proposed role in translation. Evidence from global translatome studies indicates that light regulates plant photomorphogenesis through highly dynamic and pervasive translational control [47,48,49], and CRYs are required for maintaining light-responsive translational changes [50]. Thus, the identification of the CRY1-eIF3G1 complex suggests a potential mechanistic connection between photoreceptor signaling and the translation initiation machinery. In this work, we investigated the interaction of eIF3G1 and CRY1 under light conditions, suggesting that eIF3G1 may interact with photoactivated CRY1 complexes. This raises a critical question of whether CRYs interact with eIF3G1 in a constitutive manner or through a light-dependent process. Future biochemical experiments will focus on characterizing the blue-light dependency of this interaction and elucidating how CRY1-mediated translational control. This interaction suggests a potential functional link through which plants coordinate protein synthesis with light signaling during photomorphogenesis.

The functional importance of *eIF3G1* is supported by the profound transcriptional feedback observed in our study. Although translatome profiling was not performed, the transcriptional downregulation of genes involved in translation and ribosome biogenesis upon *eIF3G1* imbalance implies its important role within the protein synthesis machinery. Specifically, we observed that both the *eif3g1* mutant and the overexpression lines exhibited a substantial proportion of DEGs changing in the same direction, and these genes were enriched in the categories related to translation regulation, photosynthesis, and stress response. This finding supports that maintaining precise *eIF3G1* levels is associated with transcriptional changes in growth- and stress-related pathways. Consistently, dosage sensitivity has also been reported for other eIF3 subunits, as both loss- and gain-of-function of *eIF3e* lead to developmental defects [51]. Such co-directional transcriptomic responses to perturbation of a single gene have been described for the heat shock factor HSF1 in *C. elegans* [52]. Together with the interaction between eIF3G1 and heat shock factor HSFB1 in *Arabidopsis* [39] and the documented role of eIF3g in mediating transcript-specific translation under heat stress in yeast [29], these observations support the observed co-directional DEG pattern and suggest eIF3G1 in stress-responsive regulation. Blue light functions as a developmental signal but can also promote photooxidative stress, requiring plants to dynamically balance growth and protection [53,54]. Thus, the CRY1-eIF3G1 complex may contribute to this balance by modulating translational efficiency or the prioritization of specific mRNA subsets, resulting in photomorphogenesis. Although the co-directional pattern of DEGs was predominant, there were still 64 DEGs showing opposite directions of transcriptional regulation. These genes were enriched in redox homeostasis, such as the cellular response to hypoxia, and are involved in peroxisome function. Consistent with the role of ROS balance in root meristem regulation [21], they may contribute to differences in root developmental phenotypes. They may therefore act as key modulators distinguishing root phenotypes between the *eif3g1* mutant and *eIF3G1* overexpression lines.

Our findings indicate that light perception in the shoot is associated with eIF3G1-related differences in primary root elongation under blue light. This observation suggests a potential role for *eIF3G1* within a systemic shoot-to-root communication process. Roots typically develop in dark soil, and their growth is strongly influenced by shoot-derived light signals transmitted via mobile factors such as HY5, auxin, and sucrose [15,16,17,18,19]. Within this context, it could be hypothesized that *eIF3G1* may either act as a phloem-mobile signal itself or function in the shoot to regulate the translation of specific mobile effectors that subsequently move to the root to modulate growth. However, whether *eIF3G1* acts as a direct systemic messenger or a localized modulator of such signals remains to be definitively determined. To distinguish between these possibilities, future studies will be required to directly test protein mobility and tissue-specific function [16].

Phenotypic analyses show that *CRY1* and *eIF3G1* function as antagonistic regulators of primary root elongation under blue light. While cytoplasmic CRY1 acts as a positive regulator [14], *eIF3G1* serves as a negative modulator, and both are dependent on shoot-perceived light signaling to show their effects on the root system. Note that the *CRY1* overexpression line has been reported to promote primary root elongation [24]. Together, these findings suggest a potential balance between CRY1 and eIF3G1 in regulating root length. We propose that CRY1 and eIF3G1 form a functional rheostat that fine-tunes root elongation by balancing light perception growth and stress signals. The transcriptomic intersection between *cry1* and *eIF3G1* overexpression lines in roots suggests shared transcriptional changes in genes related to physiological processes, including auxin signaling, photosynthetic feedback, and *BIC*-associated regulation. In particular, *BIC1* and *BIC2* were consistently downregulated in both *cry1* and *eIF3G1* overexpression lines. Previous studies have shown that *CRY1* signaling promotes the transcription of *BIC1* and *BIC2* as part of a feedback regulatory circuit, whereas BIC proteins negatively regulate CRY activity by suppressing blue-light-induced CRY oligomerization [46,55]. Therefore, although *BIC1/2* are classically described as CRY inhibitors in hypocotyls, their reduced expression in *cry1* roots does not contradict the short-root phenotype observed in the *bic1 bic2* mutant. These observations suggest a potential association between *CRY1* and *BIC1/2* in roots, but their functional relationship in root development remains unclear due to the lack of genetic epistasis evidence. It is also possible that *BIC* function differs between roots and aboveground tissues. In addition, the relationship between *eIF3G1* and *CRY1* in regulating root growth remains to be clarified, and whether they act in the same pathway or in parallel remains undetermined.

## 4. Materials and Methods

### 4.1. Plant Materials and Growth Conditions

All mutants and transgenic plants were in the Columbia-0 (Col-0) background. The T-DNA insertion line SALK_029432(*eif3g1* mutant), originally from the *Arabidopsis* Biological Resource Center (ABRC, Columbus, OH, USA), was kindly provided by Dr. Xu Zheng from Henan Agricultural University [39]. The *eIF3G1 OE#4* and *OE#8* were generated by transforming Col-0 plants with the pmACT2pro: eIF3G1-Flag-GFP construct. The *cry1* mutant and *bic1bic2* double mutant were generated in our laboratory and have been previously characterized [55].

Seeds of WT (Col-0), *eif3g1* mutant, *cry1* mutant, *OE#4*, and *OE#8* were surface-sterilized and sown on 1/2 MS medium supplemented with 1% (*w*/*v*) sucrose and solidified with 1% (*w*/*v*) agar. Plates were kept at 4 °C in darkness for 2 days for stratification and then transferred to a controlled growth chamber. Seedlings were grown under long-day conditions (16 h light/8 h dark) at 22 °C with approximately 60% relative humidity. White light was provided at an intensity of approximately 100 μmol·m^−2^·s^−1^ unless otherwise indicated.

### 4.2. RNA Isolation and Gene Cloning

Total RNA for gene cloning was extracted from 5-day-old *Arabidopsis* seedlings grown on 1/2 MS medium using the RNAprep Pure Plant Total RNA Extraction Kit (Tiangen, Beijing, China; Cat. DP432) according to the manufacturer’s instructions. Residual genomic DNA was removed according to the kit protocol. RNA integrity was examined by 1% agarose gel electrophoresis, and RNA concentration and purity were determined by spectrophotometry. Only RNA samples with appropriate quality were used for subsequent analyses. First-strand cDNA was synthesized from 1 μg of total RNA using the Revert Aid First Strand cDNA Synthesis Kit (Thermo Scientific, Waltham, MA, USA; Cat. K1691) following the manufacturer’s protocol. The resulting cDNA was diluted appropriately and used as the template for downstream gene amplification and vector construction.

### 4.3. Protein Interaction Assays

Protein–protein interactions were analyzed using Co-IP, BiFC, and split-LUC assays. Primers used for the construction of all related vectors are listed in Supplementary Table S8.

For Co-IP assays, the coding sequences of eIF3G1 and CRY1 were cloned into plant expression vectors to generate C-terminal epitope-tagged constructs (eIF3G1-Flag-GFP and 4Myc-CRY1). The indicated plasmids were introduced into *Agrobacterium tumefaciens* strain GV3101 and the agrobacteria were infiltrated into *Nicotiana benthamiana* leaves using a disposable syringe for transient co-expression. After infiltration, plants were maintained under controlled growth conditions (16 h light/8 h dark, white light at 100 μmol m^−2^ s^−1^) for 48 h. Infiltrated leaf tissues were harvested and homogenized in ice-cold extraction buffer containing 50 mM Tris-HCl (pH 8.0), 150 mM NaCl, 0.1% (*w*/*v*) PVP, 1% (*v*/*v*) Triton X-100, 1 mM EDTA, 1× protease inhibitor cocktail, 1 mM DTT, and 1 mM PMSF for 30 min at 4 °C. The homogenate was clarified by centrifugation at 12,000× *g* for 15 min at 4 °C. The supernatant was incubated with GFP-Trap Agarose beads at 4 °C for 2 h with gentle rotation. After incubation, the beads were washed at least five times with wash buffer (extraction buffer without protease inhibitor cocktail, DTT and PMSF) and eluted by boiling in SDS loading buffer. Immunoprecipitated proteins were detected by immunoblotting using anti-MYC and anti-GFP antibodies.

For the BiFC assays, the full-length coding sequences of eIF3G1 and CRY1 were fused to the N-terminal (nYFP) or C-terminal (cYFP) fragments of yellow fluorescent protein, respectively. The resulting constructs were introduced into Agrobacterium tumefaciens GV3101 and co-infiltrated into fully expanded leaves of *Nicotiana benthamiana*. After 48 h incubation under the same conditions, fluorescence signals were observed under an inverted fluorescence microscope. YFP fluorescence was excited at 514 nm and detected at 525–560 nm. At least three independent biological replicates were examined. Empty vectors fused with the Nluc sequence were used as negative controls. mCherry fused with a nuclear localization signal (NLS) served as a nuclear localization marker. All images were acquired using identical microscope settings (including laser intensity, gain, and exposure time) and processed uniformly.

For Split-LUC assays, the coding sequences of eIF3G1 and CRY1 were fused to the N-terminal (nLUC) and C-terminal (cLUC) fragments of firefly luciferase, respectively. The constructs were transformed into *Agrobacterium tumefaciens* GV3101 and co-infiltrated into *Nicotiana benthamiana* leaves. At 48 h post-infiltration under the same conditions, infiltrated leaf areas were sprayed with 1 mM D-luciferin potassium salt and incubated in darkness for 5 min. Luminescence signals were captured using a CCD imaging system with identical exposure settings across all samples. Each treatment included at least three independent biological replicates.

### 4.4. Blue-Light-Regulated Root Phenotypic Analysis

For phenotypic assays, seeds of the indicated genotypes were surface-sterilized and sown on vertically oriented 1/2 MS agar medium supplemented with 1% (*w*/*v*) sucrose. After stratification at 4 °C for 2 days in darkness, plates were transferred to growth chambers and incubated under continuous blue light for 5 days. Blue-light fluence rates of 0, 5, 10, 20, and 40 μmol m^−2^ s^−1^ were applied where indicated, while other growth conditions remained constant (22 °C).

Five-day-old seedlings were photographed using a Canon digital camera under identical acquisition settings. Primary root length and hypocotyl length were quantified from digital images using ImageJ (version 1.54f; National Institutes of Health, Bethesda, MD, USA). For each genotype and light condition, at least 18 seedlings were analyzed per biological replicate. All experiments were independently repeated at least three times with consistent results. Data from one representative experiment are presented as mean ± SD. Significant differences among genotypes within the same light condition were determined by one-way ANOVA followed by Tukey’s multiple comparison test.

### 4.5. Separate Illumination System Assays

To investigate shoot–root light signaling, a separate illumination system was established based on previously described methods [21]. Three-day-old seedlings were transferred to 10 cm square plates containing 1/2 MS medium supplemented with 1% (*v*/*v*) black ink to prevent light piping through the agar and cultured for an additional 3 days under 20 μmol m^−2^ s^−1^ blue light conditions. Seedlings were arranged horizontally, parallel to the agar surface, and custom-made aluminum foil boxes were used to shield either the shoots or the roots, generating Shoot light/Root dark and Shoot dark/Root light configurations. The efficacy of light exclusion was validated using a UPRtek PG200N spectrometer (UPRtek, Zhunan, Taiwa), which confirmed that the photosynthetic photon flux density (PPFD) within the shielded regions was <0.002 μmol m^−2^ s^−1^, indicating near-complete elimination of detectable light. Treatments with the same light intensity were conducted simultaneously within the same growth chamber (22 ± 1 °C, 60% relative humidity). After treatment, root length was measured to evaluate the effect of shoot illumination on root growth.

### 4.6. Arabidopsis Root Sampling, RNA Extraction and RNA Sequencing

*Arabidopsis thaliana* WT, *eif3g1* mutant, and *OE#4* lines were grown vertically on 1/2 MS medium under continuous blue light (20 μmol·m^−2^·s^−1^) for 5 days. Roots were harvested for subsequent analyses. For each genotype, roots from multiple seedlings were pooled to constitute one biological replicate, and three independent biological replicates were collected per genotype. Samples were immediately frozen in liquid nitrogen and ground into a fine powder using a pre-chilled mortar and pestle.

Total RNA for RNA-Seq was extracted using a Quick RNA Isolation Kit (Huayueyang, Beijing, China, Cat. BC10) according to the manufacturer’s instructions. RNA integrity and concentration were assessed prior to library construction. Sequencing libraries were prepared using VAHTS Universal V6 RNA-Seq Library Prep Kit for Illumina (Vazyme, Nanjing, China, Cat. NR606-01) and sequenced on an Illumina NovaSeq™ X Plus platform at Annoroad Gene Technology (Beijing) Co., Ltd. (Beijing, China) to generate 150 bp paired-end reads. For each library, between 19.3 to 29.0 million paired-end reads were generated.

### 4.7. RNA-Seq Data Processing, Differential Expression and PCA Analysis

Raw sequencing reads were trimmed using Trimmomatic (version 0.38) to remove adaptor sequences and low-quality bases. Clean reads were aligned to the *Arabidopsis* thaliana reference genome (TAIR10) using RSEM (version 1.2.28) with bowtie2 aligner (version 2.5.1) [56,57]. Gene-level expected counts and FPKM values were obtained from the RSEM output.

To evaluate global transcriptional variation among samples, principal component analysis (PCA) was conducted within the DESeq2 framework. Raw count data were first normalized for sequencing depth in DESeq2 using the median-of-ratios method. Variance-stabilizing transformation (VST) was applied to raw count data using the vst function in DESeq2, and PCA was performed using the plot PCA function. The first two principal components were used to assess sample clustering and reproducibility among biological replicates. Differential expression analysis was performed using the R package DESeq2 (version 1.42.1) [58]. Raw expected counts were used to construct the DESeqDataSet object with the appropriate experimental design formula. *p*-values were adjusted for multiple testing using the Benjamini–Hochberg false discovery rate (FDR) method. Genes with FDR-adjusted *p*-values < 0.05 and |log2FoldChange| ≥ log2(1.5) were defined as differentially expressed genes (DEGs).

### 4.8. Gene Ontology (GO) Enrichment Analysis

Gene Ontology (GO) enrichment analysis of DEGs was performed using the resampling method implemented in clusterProfiler (version 4.10.1) [59], which corrects for potential gene length bias in RNA-Seq data. Multiple testing correction was conducted using the Benjamini–Hochberg FDR method. GO terms with FDR-adjusted *p*-values < 0.05 were considered significantly enriched.

### 4.9. Data Visualization

All plots were generated using custom R scripts or TBtools (version 2.376) [60] or GraphPad Prism (version 10.0). Heatmaps and Venn diagrams were plotted using TBtools. For heatmap visualization, fragments per kilobase of transcript per million mapped reads (FPKM) values generated by RSEM were used.

## 5. Conclusions

Our results identify eIF3G1 as a novel cytoplasmic CRY1-interacting protein associated with the shoot-dependent root growth response under blue light. Phenotypic analyses indicate that *eIF3G1* negatively regulates primary root elongation and contributes to the control of root apical meristem size. Transcriptome analysis further showed that *eIF3G1* perturbation is associated with changes in the expression of genes related to translation and light responses. The transcriptomic overlap between cry1 mutation and eIF3G1 overexpression in roots suggests shared transcriptional responses affecting processes such as auxin signaling, photosynthesis-related pathways, and BIC-associated regulation. These findings suggest that eIF3G1 may be associated with CRY1-related light signaling and root growth responses.

## Data Availability

Illumina sequencing data have been deposited in the Genome Sequence Archive [61] at the National Genomics Data Center [62], China National Center for Bioinformation/Beijing Institute of Genomics, Chinese Academy of Sciences (GSA: CRA039529 and CRA039531) and are publicly accessible at https://ngdc.cncb.ac.cn/gsa (accessed on 1 April 2026).

## Supplementary Materials

The following supporting information can be downloaded at: https://www.mdpi.com/article/10.3390/plants15111682/s1, Figure S1: Immunoblot analysis of eIF3G1 protein accumulation in transgenic *Arabidopsis* lines; Figure S2: Subcellular localization of eIF3G1 in *Arabidopsis* root tips; Figure S3: Uncropped immunoblot corresponding to Figure 1b; Figure S4: Genotyping of the *eif3g1* (SALK_029432) T-DNA insertion lines; Figure S5: PCA analysis of RNA-Seq data principal component analysis; Figure S6: Gene Ontology enrichment analysis of genes coordinately regulated in *eif3g1* and *OE* lines; Figure S7: Volcano plot of differentially expressed genes (DEGs) from RNA-Seq; Table S1: Differentially expressed genes (DEGs) identified in *eif3g1* compared with WT using DESeq2; Table S2: Differentially expressed genes (DEGs) identified in *eIF3G1*-OE compared with WT using DESeq2; Table S3: Sixty-four genes exhibiting opposite expression changes between the *eif3g1* mutant and overexpression lines; Table S4: Differentially expressed genes (DEGs) identified in *cry1* compared with WT using DESeq2; Table S5: Three hundred and sixteen genes consistently regulated in the *cry1* mutant and *eIF3G1* overexpression lines relative to WT; Table S6: GO enrichment of 133 genes consistently downregulated in the *cry1* mutant and *eIF3G1* overexpression lines relative to the wild type (WT); Table S7: GO enrichment of 183 genes consistently upregulated in the *cry1* mutant and *eIF3G1* overexpression lines relative to the wild type (WT); Table S8: List of primers and their sequences.
