# Supplementary material for: A CRY1 Interactor eIF3G1 Negatively Regulates Root Growth Under Blue Light in Arabidopsis
Source: Plants (Basel). 2026 May 29;15(11):1682. doi: 10.3390/plants15111682 (PMC13258889; doi:10.3390/plants15111682)
Supplement: Supplementary file 1 [file plants-15-01682-s001.zip › plants-4266533-supplementary/Supplementary Figure.pdf]

Supplementary Figures

Chen *et al.*

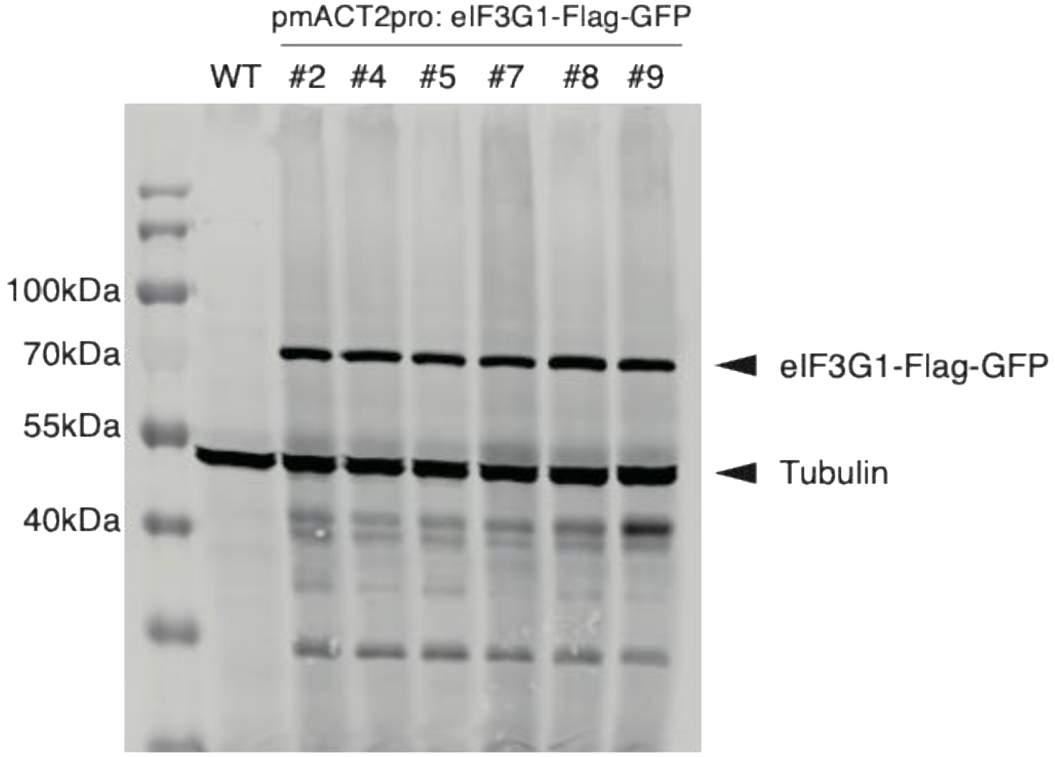

Figure S1. Immunoblot analysis of eIF3G1 protein accumulation in transgenic *Arabidopsis* lines.

Total proteins extracted from 5-day-old seedlings of wild-type and independent transgenic lines expressing *PmACT2:EGFP-EIF3G1* are subjected to immunoblot analysis using an anti-GFP antibody. A specific EGFP-EIF3G1 fusion protein band is detected at approximately 70 kDa in transgenic lines (#2, #4, #5, #7, #8, and #9), but not in the wild-type control (WT). Immunoblot detection using an anti-Tubulin antibody is performed as a loading control. The presented blot is uncropped.

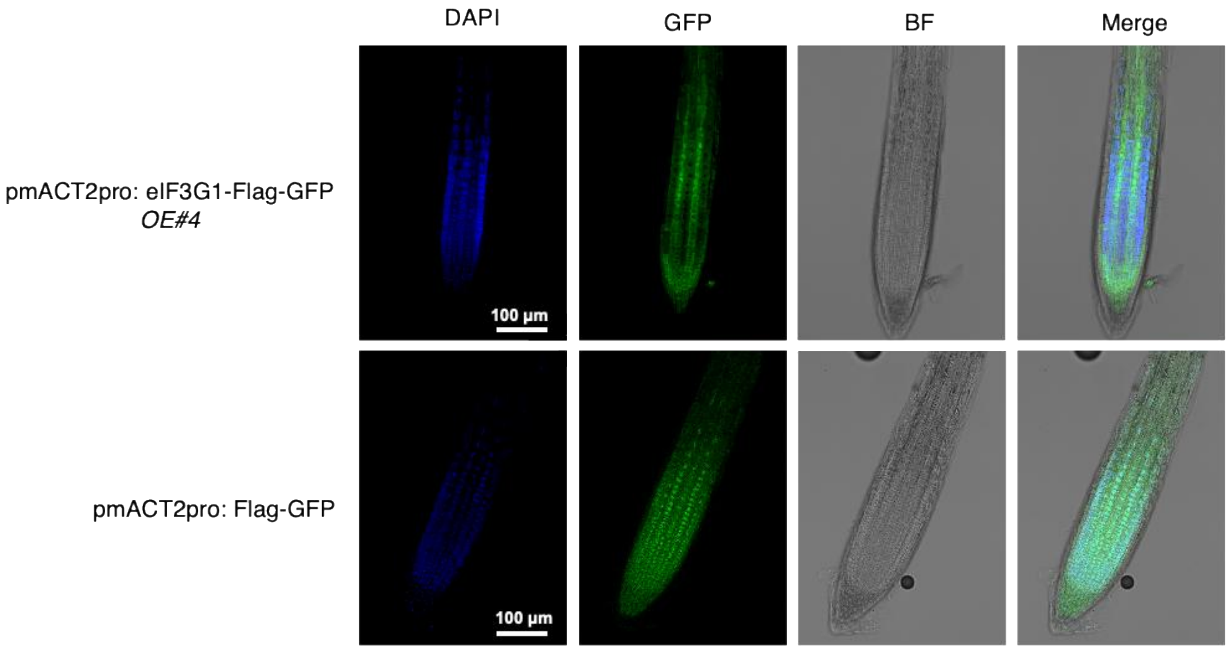

Figure S2. Subcellular localization of eIF3G1 in *Arabidopsis* root tips. Representative fluorescence microscopy images of root tips from transgenic plants expressing eIF3G1-Flag-GFP (OE#4) or the Flag-GFP empty vector control under the ACT2 promoter (pmACT2pro). Nuclei were stained with DAPI (blue). Green signals in the GFP channel denote the eIF3G1-GFP fusion protein or free GFP-Flag protein. BF (bright-field) images illustrate cellular and tissue structures. Merged images combine DAPI, GFP and bright-field signals. Scale bars = 100 μm.

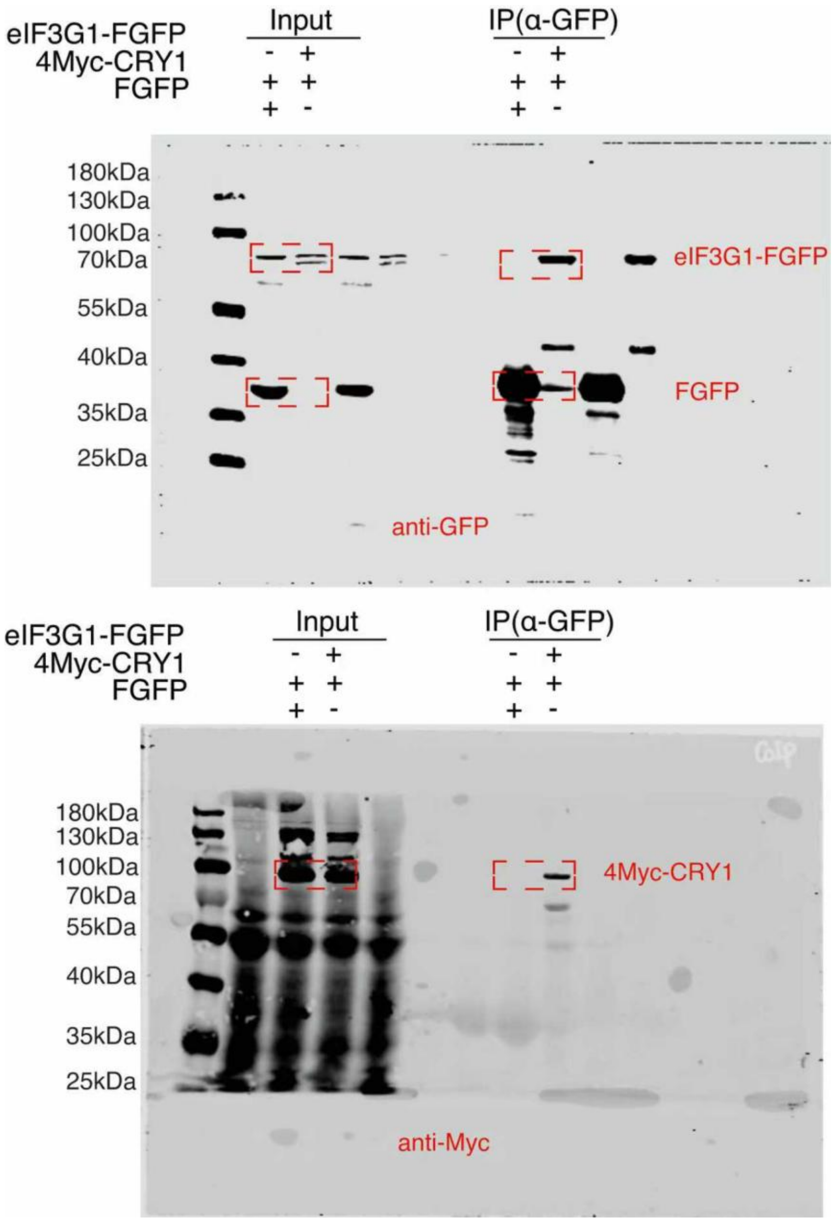

Figure S3. Uncropped immunoblot corresponding to Figure 1b.

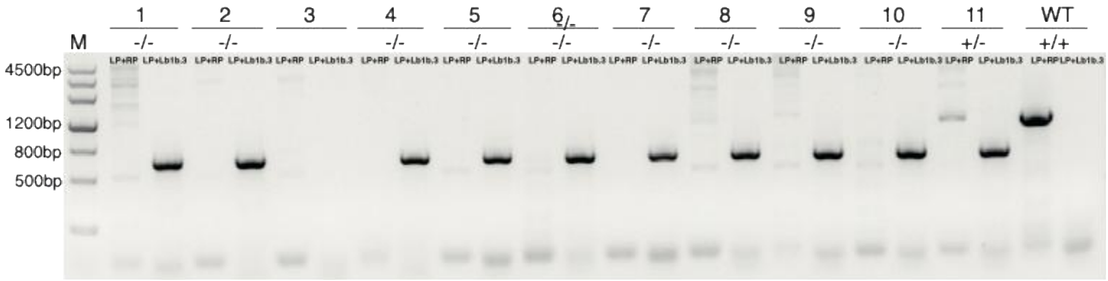

Figure S4. Genotyping of the *eif3g1* (SALK_029432) T-DNA insertion lines. The genotypes of individuals 1-11 screened from the seeds provided by the donor are determined by PCR using gene-specific primers and the T-DNA left border primer Lb1b.3 (primer information is provided in Supplementary Table 8). For each individual, the LP + RP primer pair is used to detect the wild-type allele, whereas the LP + Lb1b.3 primer pair is used to detect the T-DNA insertion allele. The LP + RP primer pair amplified a 1107 bp wild-type fragment, while LP + Lb1b.3 amplified an approximately 600 bp T-DNA insertion fragment. Individuals 1, 2, 4, 5, 6, 7, 8, 9, and 10 are identified as homozygous mutants (-/-), whereas individual 11 is identified as heterozygous (+/-). No amplification is detected for individual 3. WT, wild-type control; M, DNA molecular weight marker. Seeds descended from a single homozygous mutant plant are subsequently propagated and used for further experiments.

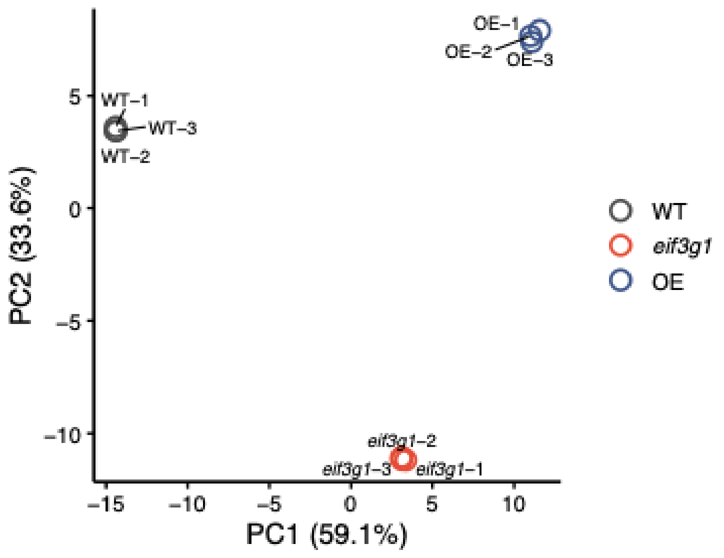

Figure S5. PCA analysis of RNA-seq Data Principal component analysis (PCA) is performed using normalized transcript abundance values from RNA-seq datasets. PC1 and PC2 explain 59.1% and 33.6% of the total variance, respectively. Samples from Col4, eif3g1, and eIF3G1 overexpression (OE) plants form distinct clusters, indicating genotype-dependent transcriptional divergence. The distribution of samples along PC1 suggests a dosage-associated gradient of transcriptional change, whereas separation along PC2 further distinguishes OE from eif3g1 lines.

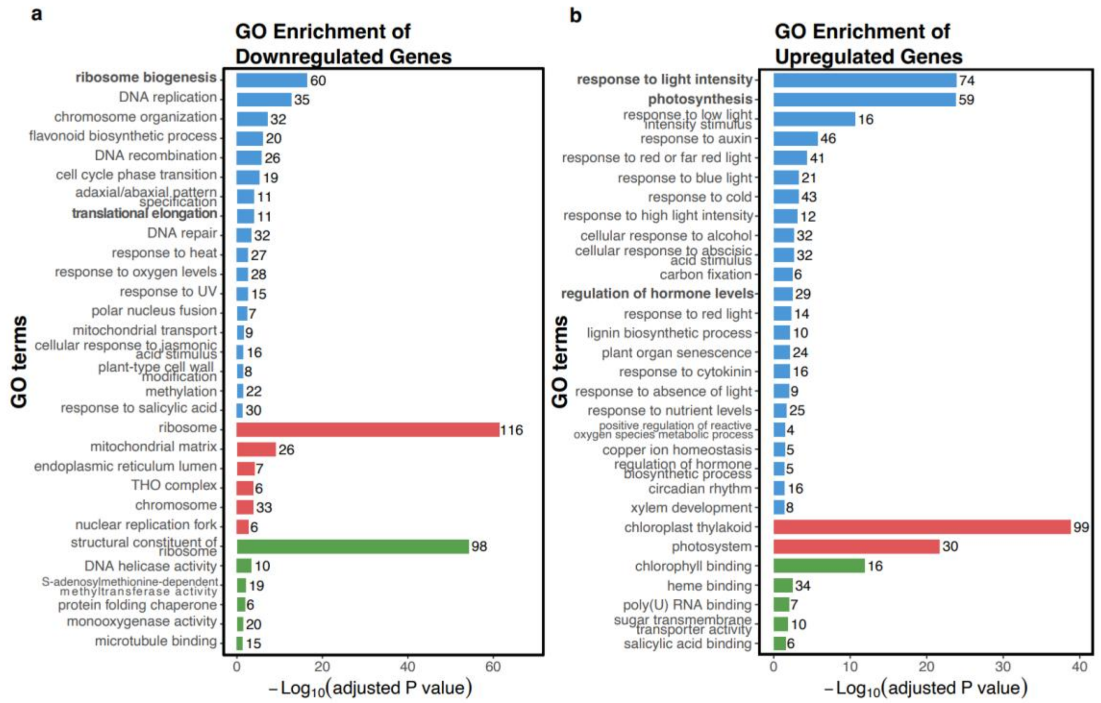

Figure S6. Gene Ontology enrichment analysis of genes coordinately regulated in eif3g1 and OE lines. (a) GO terms enriched among genes commonly downregulated in both genotypes are predominantly associated with ribosome biogenesis, translational processes, DNA replication, chromatin organization, and cell cycle progression. (b) GO terms enriched among genes commonly upregulated in both genotypes include light-responsive pathways, photosynthesis-related processes, hormone signaling, and stress-associated responses. Bar plots represent significantly enriched GO terms ranked by -log10(adjusted p value).

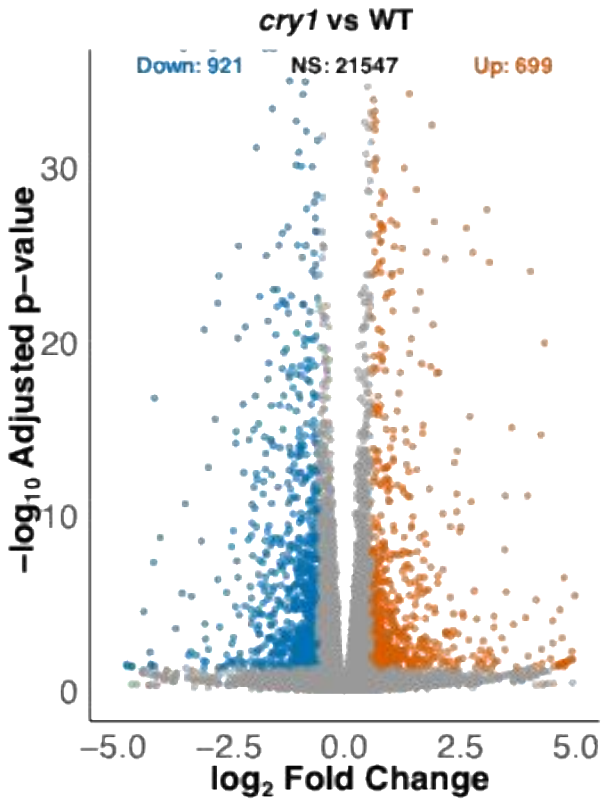

Figure S7. Volcano plot of differentially expressed genes (DEGs) from RNA-seq analysis comparing the cry1 mutant with wild type (WT) under blue light.
